# Supplementary material for: Gamma, E-Beam and X-ray Irradiations on PE/EVOH/PE Multilayer Film: An Industrial Point of View Regarding the Impact on Mechanical Properties
Source: Polymers (Basel). 2023 Jun 24;15(13):2799. doi: 10.3390/polym15132799 (PMC10346571; doi:10.3390/polym15132799)
Supplement: Supplementary file 1 [file polymers-15-02799-s001.zip › polymers-2343453-supplementary.pdf]

Supporting information

Gamma, E-Beam and X-ray Irradiations on PE/EVOH/PE Multilayer Film: An Industrial Point of View Regarding the Impact on Mechanical Properties

Nina Girard-Perier [1,*], Sylvain R. A. Marque [2], Nathalie Dupuy [3], Blanche Krieguer [1,2,3] and Samuel Dorey [1,*]

[1] Sartorius Stedim FMT S.A.S, Z.I. Les Paluds, Avenue de Jouques CS91051, CEDEX, 13781 Aubagne, France; blanche.krieguer@sartorius.com

[2] Aix Marseille Univ, CNRS, ICR, Case 551, 13397 Marseille, France; sylvain.marque@univ-amu.fr

[3] Aix Marseille Univ, Avignon Université, CNRS, IRD, IMBE, 13013 Marseille, France; nathalie.dupuy@univ-amu.fr

* Correspondence: nina.perier@sartorius.com (N.G.-P.); samuel.dorey@sartorius.com (S.D.)

Summary of table of contents:

Table S1: Raw data of the mechanical properties obtained by tensile test on PE/EVOH/PE film (TD); Table S2: Raw data of the mechanical properties obtained by tensile test on PE/EVOH/PE film (MD); Table S3: T-test summary. p-values obtained for UTS MD a), UTS TD b), Elongation MD c) and Elongation TD d); Table S4: Equivalence test summary. Null hypothesis: Ratio≤0.75 or Ratio≥1.25. Alternative hypothesis: 0.75<Ratio<1.25. p-values obtained for UTS MD a), UTS TD b), Elongation MD c) and Elongation TD d); Table S5: Raw data of the thermal transitions obtained by DMA on PE/EVOH/PE film (TD); Table S6: Raw data of the thermal transitions obtained by DMA on PE/EVOH/PE film (MD); Figure S1: Example of DMA curves of PE/EVOH/PE in TD (1 batch, 1 specimen); Table S7: T-test summary. p-values obtained for γ transition MD a), γ transition TD b), β transition MD c) and β transition TD d); Table S8: Equivalence test summary. Null hypothesis: Difference≤-5 or Difference≥5. Alternative hypothesis: -5<Difference<5. p-values obtained for γ transition MD a), γ transition TD b), β transition MD c), β transition TD d)

Table S1: Raw data of the mechanical properties obtained by tensile test on PE/EVOH/PE film (TD)

	Irradiation modality (#Batch)	S80 Transversal Direction (TD)						
		Sample 1	Sample 2	Sample 3	Sample 4	Sample 5	Sample 6	Sample 7
Elongation at Fmax (%)	Gamma 50kGy Batch 2	497	501	488	462	506	491	-
	X-ray 50kGy Batch 3	515	485	508	519	524	527	-
	Gamma 50kGy Batch 2	502	487	484	487	492	488	-
	X-ray 50kGy Batch 2	479	490	478	476	461	495	-
	Non sterile Batch 2	522	548	538	540	545	546	-
	E-beam 50kGy Batch 4	461	483	474	461	459	465	477
	E-beam 50kGy Batch 5	448	455	455	449	474	454	458
Ultimate Tensile Strength (MPa)	Gamma 50kGy Batch 1	15.6	15.5	15.2	14.3	15.9	15.4	-
	X-ray 50kGy Batch 3	16.0	15.3	16.0	16.1	16.1	16.6	-
	Gamma 50kGy Batch 2	15.6	15.4	15.4	15.1	15.2	15.2	-
	X-ray 50kGy Batch 2	15.7	15.8	15.9	15.8	15.3	16.0	-
	Non sterile Batch 2	16.9	17.6	17.2	17.8	17.8	17.6	-
	E-beam 50kGy Batch 4	17.1	17.8	17.9	17.1	16.8	17.5	17.4
	E-beam 50kGy Batch 4	16.4	16.4	16.3	16.0	17.3	16.7	16.6

Table S1: Raw data of the mechanical properties obtained by tensile test on PE/EVOH/PE film (MD)

	Irradiation modality	S80 Machine Direction (MD) Sample 1	Sample 2	Sample 3	Sample 4	Sample 5	Sample 6	Sample 7	Sample 8
Elongation at Fmax (%)	Gamma 50kGy Batch 1	432	428	438	441	442	427	-	-
	X-ray 50kGy Batch 3	439	456	452	458	448	429	-	-
	Gamma 50kGy Batch 2	392	408	409	388	395	406	-	-
	X-ray 50kGy Batch 2	437	422	410	430	421	416	-	-
	Non sterile Batch 2	422	423	409	413	415	404	-	-
	E-beam 50kGy Batch 4	416	406	393	448	413	411	406	389
	E-beam 50kGy Batch 5	437	434	437	418	403	437	442	413
Ultimate Tensile Strength (MPa)	Gamma 50kGy Batch 1	16.9	16.8	17.3	17.0	17.0	16.9	-	-
	X-ray 50kGy Batch 3	17.4	17.8	18.2	18.3	17.4	17.1	-	-
	Gamma 50kGy Batch 2	16.1	16.6	16.6	16.1	16.4	16.4	-	-
	X-ray 50kGy Batch 2	18.2	17.2	17.0	17.5	17.5	17.1	-	-
	Non sterile Batch 2	17.4	16.9	17.0	17.1	17.5	16.7	-	-
	E-beam 50kGy Batch 4	19.1	18.5	17.9	20.3	18.8	19.7	18.9	18.0
	E-beam 50kGy Batch 5	19.7	19.3	19.5	18.9	18.2	19.4	19.4	18.7

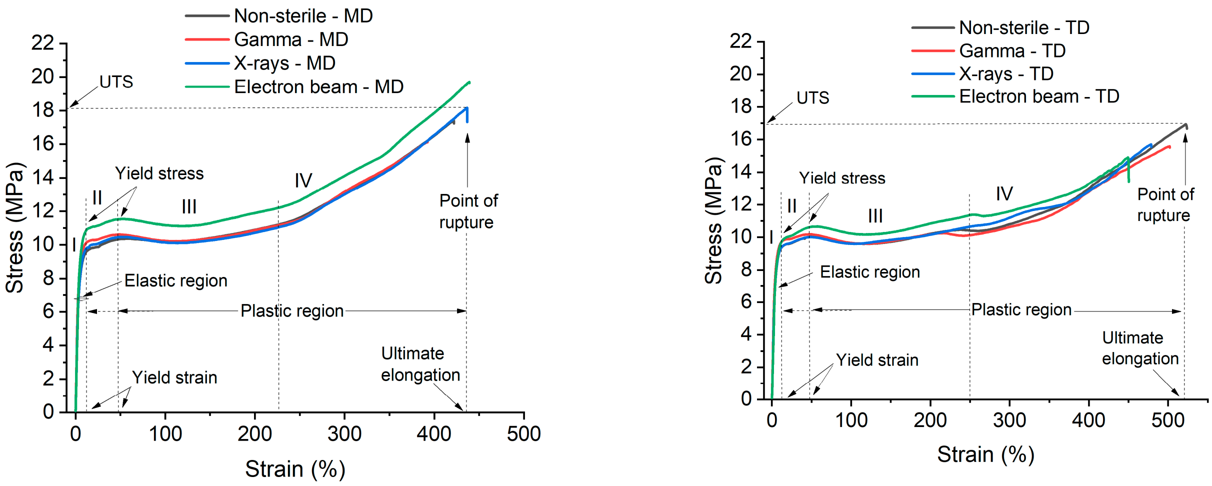

(**a**) (**b**)

Figure S1: Tensile curves of PE/EVOH/PE in MD a) and TD b) (1 batch, 1 specimen for each direction) – non sterile in black, gamma irradiated sample in red, X-rays irradiated sample in blue, Electron beam irradiated sample in green. 50+/-5 KGy use for all technologies. UTS and Ultimate elongation values are used for Figure 3 and 4.

Ultimate tensile strength at break and ultimate elongation are defined as the maximum strength and maximum elongation which the material can withstand (ISO 527-1, 2019). Yield strength (or strain) is the strength (or strain) level at which plastic deformation initiates. Importantly, in this section, regarding the EVA and PE bags, the seal part is never damaged during the mechanical test. This means that only the film part is broken by the strain applied (Fig. 3). As observed in Fig. 3b-d, the seal shows no opening or tear and no delamination takes place. Once the interpenetration of the polymer chains has occurred, the seal then keeps its function of sealing, whatever the gamma irradiation dose and the seal location.

*Table S2: T-test summary. p-values obtained for UTS MD a), UTS TD b), Elongation MD c) and Elongation TD d). * means that the difference between 2 values is statistically significant and p-value <5%. ** means that the difference between 2 values is statistically significant and p-value <1%.*

UTS MD	Non-sterile	Gamma	X-rays	E-beam
Non-sterile		* P=0.0288	* P=0.0439	** P=4.10E-7
Gamma	* P=0.0288		** P=3.72E-5	** P=7.47E-12
X-rays	* P=0.0439	** P=3.72E-5		** P=1.70E-7

E-beam	**<br>P=4.10E-7	**<br>P=7.47E-12	**<br>P=1.70E-7	

a)

UTS TD	Non-sterile	Gamma	X-rays	E-beam
Non-sterile		**<br>P=4.49E-9	**<br>P=1.17E-7	Non significant
Gamma	**<br>P=4.49E-9		**<br>P=0.00117	**<br>P=2.12E-8
X-rays	**<br>P=1.17E-7	**<br>P=0.00117		**<br>P=1.30E-5
E-beam	Non significant	**<br>P=2.12E-8	**<br>P=1.30E-5	

b)

Elong MD	Non-sterile	Gamma	X-rays	E-beam
Non-sterile		Non significant	**<br>P=0.00979	Non significant
Gamma	Non significant		*<br>P=0.0254	Non significant
X-rays	**<br>P=0.00979	*<br>P=0.0254		*<br>P=0.0243
E-beam	Non significant	Non significant	*<br>P=0.0243	

c)

Elong TD	Non-sterile	Gamma	X-rays	E-beam

Non-sterile		** P=9.49E-8	** P=2.78E-4	** P=1.11E-11
Gamma	** P=9.49E-8		Non significant	** P=1.13E-6
X-rays	** P=2.78E-4	Non significant		** P=2.54E-5
E-beam	** P=1.11E-11	** P=1.13E-6	** P=2.54E-5	

d)

Table S3: Equivalence test summary. Null hypothesis: Ratio≤0.75 or Ratio≥1.25. Alternative hypothesis: 0.75<Ratio<1.25. p-values obtained for UTS MD a), UTS TD b), Elongation MD c) and Elongation TD d) ✓*: equivalence*

Test results:

Test

Null hypothesis: Ratio ≤ 0,65 or Ratio ≥ 1,35
Alternative hypothesis: 0,65 < Ratio < 1,35
α level: 0,05

Null Hypothesis	DF	T-Value	P-Value
Ratio ≤ 0,65	24	45,464	0,000
Ratio ≥ 1,35	24	-15,821	0,000

The greater of the two P-Values is 0,000. Can claim equivalence.

p-values:

UTS MD	Non-sterile	Gamma	X-rays	E-beam
Non-sterile		✓ Ratio ≤0.65: 0.000 Ratio ≥1.35: 0.000	✓ Ratio ≤0.65: 0.000 Ratio ≥1.35: 0.000	✓ Ratio ≤0.65: 0.000 Ratio ≥1.35: 0.000
Gamma	✓ Ratio ≤0.65: 0.000		✓ Ratio ≤0.65: 0.000	✓ Ratio ≤0.65: 0.000

	Ratio ≥1.35: 0.000		Ratio ≥1.35: 0.000	Ratio ≥1.35: 0.000
X-rays	✓<br>Ratio ≤0.65: 0.000<br>Ratio ≥1.35: 0.000	✓<br>Ratio ≤0.65: 0.000<br>Ratio ≥1.35: 0.000		✓<br>Ratio ≤0.65: 0.000<br>Ratio ≥1.35: 0.000
E-beam	✓<br>Ratio ≤0.65: 0.000<br>Ratio ≥1.35: 0.000	✓<br>Ratio ≤0.65: 0.000<br>Ratio ≥1.35: 0.000	✓<br>Ratio ≤0.65: 0.000<br>Ratio ≥1.35: 0.000	

a)

UTS TD	Non-sterile	Gamma	X-rays	E-beam
Non-sterile		✓<br>Ratio ≤0.65: 0.000<br>Ratio ≥1.35: 0.000	✓<br>Ratio ≤0.65: 0.000<br>Ratio ≥1.35: 0.000	✓<br>Ratio ≤0.65: 0.000<br>Ratio ≥1.35: 0.000
Gamma	✓<br>Ratio ≤0.65: 0.000<br>Ratio ≥1.35: 0.000		✓<br>Ratio ≤0.65: 0.000<br>Ratio ≥1.35: 0.000	✓<br>Ratio ≤0.65: 0.000<br>Ratio ≥1.35: 0.000
X-rays	✓<br>Ratio ≤0.65: 0.000<br>Ratio ≥1.35: 0.000	✓<br>Ratio ≤0.65: 0.000<br>Ratio ≥1.35: 0.000		✓<br>Ratio ≤0.65: 0.000<br>Ratio ≥1.35: 0.000
E-beam	✓<br>Ratio ≤0.65: 0.000<br>Ratio ≥1.35: 0.000	✓<br>Ratio ≤0.65: 0.000<br>Ratio ≥1.35: 0.000	✓<br>Ratio ≤0.65: 0.000<br>Ratio ≥1.35: 0.000	

b)

Elong MD	Non-sterile	Gamma	X-rays	E-beam

Non-sterile		✓<br>Ratio ≤0.75: 0.000<br>Ratio ≥1.25: 0.000	✓<br>Ratio ≤0.75: 0.000<br>Ratio ≥1.25: 0.000	✓<br>Ratio ≤0.75: 0.000<br>Ratio ≥1.25: 0.000
Gamma	✓<br>Ratio ≤0.75: 0.000<br>Ratio ≥1.25: 0.000		✓<br>Ratio ≤0.75: 0.000<br>Ratio ≥1.25: 0.000	✓<br>Ratio ≤0.75: 0.000<br>Ratio ≥1.25: 0.000
X-rays	✓<br>Ratio ≤0.75: 0.000<br>Ratio ≥1.25: 0.000	✓<br>Ratio ≤0.75: 0.000<br>Ratio ≥1.25: 0.000		✓<br>Ratio ≤0.75: 0.000<br>Ratio ≥1.25: 0.000
E-beam	✓<br>Ratio ≤0.75: 0.000<br>Ratio ≥1.25: 0.000	✓<br>Ratio ≤0.75: 0.000<br>Ratio ≥1.25: 0.000	✓<br>Ratio ≤0.75: 0.000<br>Ratio ≥1.25: 0.000	

c)

Elong TD	Non-sterile	Gamma	X-rays	E-beam
Non-sterile		✓<br>Ratio ≤0.75: 0.000<br>Ratio ≥1.25: 0.000	✓<br>Ratio ≤0.75: 0.000<br>Ratio ≥1.25: 0.000	✓<br>Ratio ≤0.75: 0.000<br>Ratio ≥1.25: 0.000
Gamma	✓<br>Ratio ≤0.75: 0.000<br>Ratio ≥1.25: 0.000		✓<br>Ratio ≤0.75: 0.000<br>Ratio ≥1.25: 0.000	✓<br>Ratio ≤0.75: 0.000<br>Ratio ≥1.25: 0.000
X-rays	✓<br>Ratio ≤0.75: 0.000<br>Ratio ≥1.25: 0.000	✓<br>Ratio ≤0.75: 0.000<br>Ratio ≥1.25: 0.000		✓<br>Ratio ≤0.75: 0.000<br>Ratio ≥1.25: 0.000
E-beam	✓<br>Ratio ≤0.75: 0.000	✓<br>Ratio ≤0.75: 0.000	✓<br>Ratio ≤0.75: 0.000	

	Ratio ≥1.25: 0.000	Ratio ≥1.25: 0.000	Ratio ≥1.25: 0.000	

d)

Table S4: Raw data of the thermal transitions obtained by DMA on PE/EVOH/PE film (TD)

Irradiation modality	Samples	Relaxation 1<br>Max Tan Delta (°C)	Relaxation 2<br>Max E" (°C)
Non sterile<br>Batch 7	Sample 1	-121.9	-23.8
	Sample 2	-116.7	-23.7
	Sample 3	-117.6	-22.3
	Sample 4	-119.4	-21.9
	Sample 5	-116.3	-23.7
	Sample 6	-118.4	-21.9
E-beam 50 kGy<br>Batch 7	Sample 1	-116	-21.8
	Sample 2	-117.9	-20.7
	Sample 3	-118	-20.3
Gamma 50kGy<br>Batch 3	Sample 1	-118.4	-21.5
	Sample 2	-116.2	-22.7
	Sample 3	-116.2	-22.7
	Sample 4	-120.0	-24.4
	Sample 5	-118.0	-24.2
X-ray 50kGy<br>Batch 6	Sample 1	-119.8	-20.4
	Sample 2	-119.8	-21.8
	Sample 3	-118.0	-21.7
	Sample 4	-118.5	-22.7
	Sample 5	-119.6	-22.8
	Sample 6	-120.4	-23.5
Gamma 50kGy<br>Batch 2	Sample 1	-122.2	-23.0
	Sample 2	-123.5	-21.8
X-ray 50kGy<br>Batch 2	Sample 1	-118.6	-21.1
	Sample 2	-119.7	-22.9

Table S5: Raw data of the thermal transitions obtained by DMA on PE/EVOH/PE film (MD)

Irradiation modality	Samples	Relaxation 1<br>Max Tan Delta (°C)	Relaxation 2<br>Max E" (°C)
Non sterile<br>Batch 7	Sample 1	-115.7	-23.2
	Sample 2	-119.6	-22.4
	Sample 3	-117.9	-21.3
	Sample 4	-117.1	-23.1
	Sample 5	-119.1	-21.4
	Sample 6	-115.9	-23.3
E-beam 50 kGy<br>Batch 7	Sample 1	-118.1	-21.2
	Sample 2	-115.5	-19.5
	Sample 3	-117.2	-19.7
	Sample 4	-115.3	-20.3
	Sample 5	-116	-22.3
	Sample 6	-117.1	-20.9
Gamma 50kGy<br>Batch 3	Sample 1	-118.3	-23.7
	Sample 2	-120.5	-24.1
	Sample 3	-119.5	-21.7
	Sample 4	-119.0	-22.7
	Sample 5	-120.9	-24.2
X-ray 50kGy<br>Batch 6	Sample 1	-121.3	-25.4
	Sample 2	-120.4	-25.2
	Sample 3	-119.9	-21.1
	Sample 4	-122.0	-21.4
	Sample 5	-119.7	-24.4
Gamma 50kGy<br>Batch 2	Sample 1	-119.6	-23.4
	Sample 2	-120.2	-21.8
X-ray 50kGy<br>Batch 2	Sample 1	-118.3	-22.5
	Sample 2	-119.6	-22.1

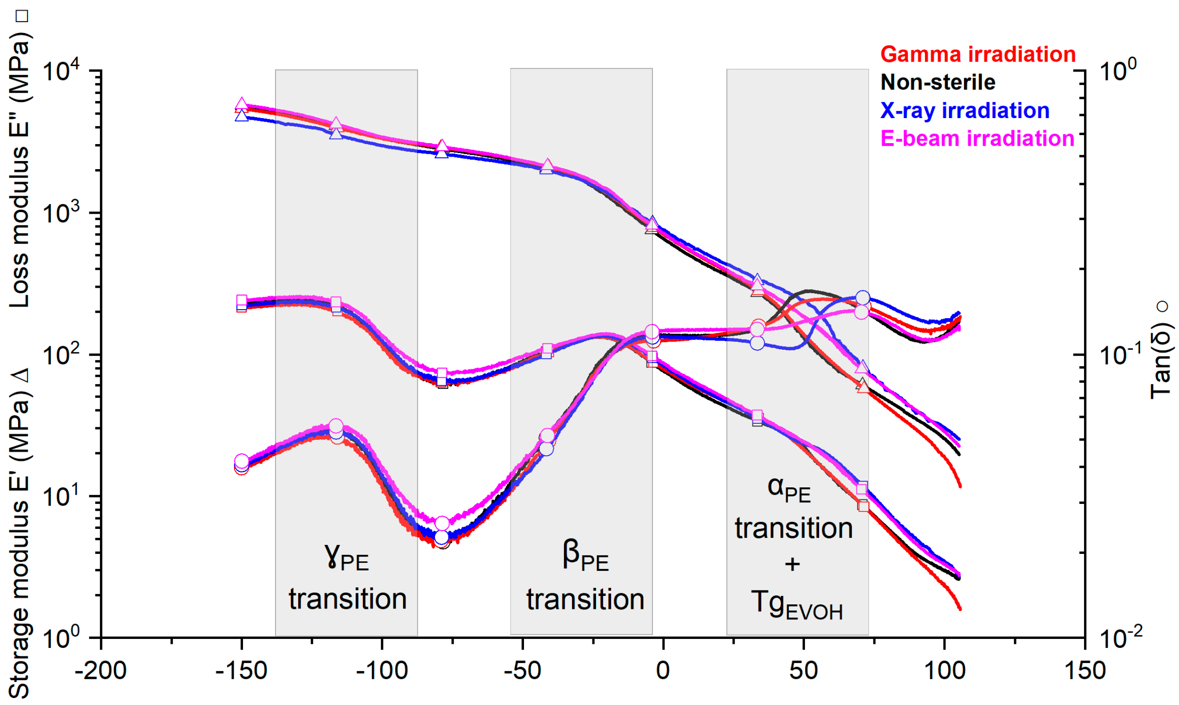

Figure S2: Example of DMA curves of PE/EVOH/PE in TD (1 batch, 1 specimen). Δ for Storage modulus E', □ for Loss modulus E" and ○ for tan δ Gamma irradiation curves are red, non-sterile are black, X-rays are blue and electron beam are pink. Gamma irradiation curves are red, non-sterile are black, X-rays are blue and electron beam are pink.

*Table S6: T-test summary. p-values obtained for γtransition MD a), γtransition TD b), β transition MD c) and β transition TD d). * means that the difference between 2 values is statistically significant and p-value <5%. ** means that the difference between 2 values is statistically significant and p-value <1%.*

γ transition MD	Non-sterile	Gamma	X-rays	E-beam
Non-sterile		* P=0.0111	** P=0.00657	Non significant
Gamma	* P=0.0111		Non significant	** P=1.31^{E}-4
X-rays	** P=0.00657	Non significant		** P=1.55^{E}-4
E-beam	Non significant	** P=1.31^{E}-4	** P=1.55^{E}-4	

a)

γ transition TD	Non-sterile	Gamma	X-rays	E-beam
Non-sterile		Non significant	Non significant	Non significant
Gamma	Non significant		Non significant	Non significant
X-rays	Non significant	Non significant		* P=0.0147
E-beam	Non significant	Non significant	* P=0.0147	

b)

β transition MD	Non-sterile	Gamma	X-rays	E-beam
Non-sterile		Non significant	Non significant	** P=0.00970
Gamma	Non significant		Non significant	** P=0.00146
X-rays	Non significant	Non significant		* P=0.0124
E-beam	** P=0.00970	** P=0.00146	* P=0.0124	

c)

β transition TD	Non-sterile	Gamma	X-rays	E-beam
Non-sterile		Non significant	Non significant	* P=0.0187

Gamma	Non significant		Non significant	* P=0.0239
X-rays	Non significant	Non significant		Non significant
E-beam	* P=0.0187	* P=0.0239	Non significant	

d)

Table S7: Equivalence test summary. Null hypothesis: Difference≤-5 or Difference≥5. Alternative hypothesis: -5<Difference<5. p-values obtained for γtransition MD a), γtransition TD b), βtransition MD c), βtransition TD d) ✓: equivalence

γtransition MD	Non-sterile	Gamma	X-rays	E-beam
Non-sterile		✓ Difference≤-5: 0.003 Difference≥5: 0.000	✓ Difference≤-5: 0.008 Difference≥5: 0.000	✓ Difference≤-5: 0.000 Difference≥5: 0.001
Gamma	✓ Difference≤-5: 0.003 Difference≥5: 0.000		✓ Difference≤-5: 0.000 Difference≥5: 0.000	✓ Difference≤-5: 0.000 Difference≥5: 0.005
X-rays	✓ Difference≤-5: 0.008 Difference≥5: 0.000	✓ Difference≤-5: 0.000 Difference≥5: 0.000		✓ Difference≤-5: 0.000 Difference≥5: 0.030
E-beam	✓ Difference≤-5: 0.000 Difference≥5: 0.001	✓ Difference≤-5: 0.000 Difference≥5: 0.005	✓ Difference≤-5: 0.000 Difference≥5: 0.030	

a)

γtransition TD	Non-sterile	Gamma	X-rays	E-beam

Non-sterile		✓<br>Difference≤-5: 0.007<br>Difference≥5: 0.001	✓<br>Difference≤-5: 0.002<br>Difference≥5: 0.000	✓<br>Difference≤-5: 0.001<br>Difference≥5: 0.005
Gamma	✓<br>Difference≤-5: 0.007<br>Difference≥5: 0.001		✓<br>Difference≤-5: 0.002<br>Difference≥5: 0.002	✓<br>Difference≤-5: 0.000<br>Difference≥5: 0.024
X-rays	✓<br>Difference≤-5: 0.002<br>Difference≥5: 0.000	✓<br>Difference≤-5: 0.002<br>Difference≥5: 0.002		✓<br>Difference≤-5: 0.005<br>Difference≥5: 0.026
E-beam	✓<br>Difference≤-5: 0.001<br>Difference≥5: 0.005	✓<br>Difference≤-5: 0.000<br>Difference≥5: 0.024	✓<br>Difference≤-5: 0.005<br>Difference≥5: 0.026	

b)

β transition MD	Non-sterile	Gamma	X-rays	E-beam
Non-sterile		✓<br>Difference≤-5: 0.000<br>Difference≥5: 0.000	✓<br>Difference≤-5: 0.000<br>Difference≥5: 0.000	✓<br>Difference≤-5: 0.000<br>Difference≥5: 0.000
Gamma	✓<br>Difference≤-5: 0.000<br>Difference≥5: 0.000		✓<br>Difference≤-5: 0.000<br>Difference≥5: 0.000	✓<br>Difference≤-5: 0.000<br>Difference≥5: 0.001
X-rays	✓<br>Difference≤-5: 0.000<br>Difference≥5: 0.000	✓<br>Difference≤-5: 0.000<br>Difference≥5: 0.000		✓<br>Difference≤-5: 0.000<br>Difference≥5: 0.006
E-beam	✓<br>Difference≤-5: 0.000	✓<br>Difference≤-5: 0.000	✓<br>Difference≤-5: 0.000	

	Difference≥5: 0.000	Difference≥5: 0.001	Difference≥5: 0.006	

c)

β transition TD	Non-sterile	Gamma	X-rays	E-beam
Non-sterile		✓ Difference≤-5: 0.000 Difference≥5: 0.000	✓ Difference≤-5: 0.000 Difference≥5: 0.000	✓ Difference≤-5: 0.000 Difference≥5: 0.003
Gamma	✓ Difference≤-5: 0.000 Difference≥5: 0.000		✓ Difference≤-5: 0.000 Difference≥5: 0.000	✓ Difference≤-5: 0.000 Difference≥5: 0.002
X-rays	✓ Difference≤-5: 0.000 Difference≥5: 0.000	✓ Difference≤-5: 0.000 Difference≥5: 0.000		✓ Difference≤-5: 0.000 Difference≥5: 0.001
E-beam	✓ Difference≤-5: 0.000 Difference≥5: 0.003	✓ Difference≤-5: 0.000 Difference≥5: 0.002	✓ Difference≤-5: 0.000 Difference≥5: 0.001	

d)
